# Synthesis of Poly(acrylic acid)-Cysteine-Based Hydrogels with Highly Customizable Mechanical Properties for Advanced Cell Culture Applications

**DOI:** 10.1021/acsomega.1c03408

**Published:** 2022-03-11

**Authors:** Sharon O. Bolanta, Sigita Malijauskaite, Kieran McGourty, Emmet J. O’Reilly

**Affiliations:** † Department of Chemical Sciences, Bernal Institute University of Limerick, Limerick V94 T9PX, Ireland; ‡ Health Research Institute (HRI), University of Limerick, Limerick V94 T9PX, Ireland

## Abstract

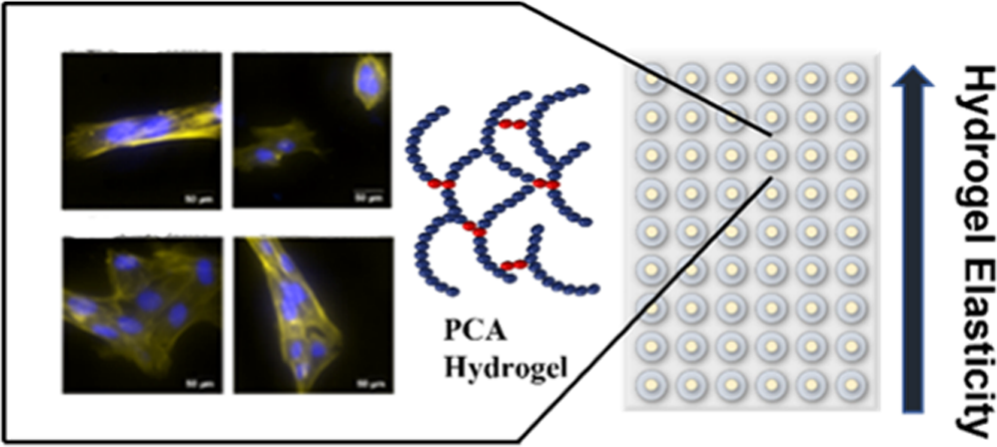

The fabrication of highly customizable scaffolds is a key enabling technology in the development of predictive *in vitro* cell models for applications in drug discovery, cancer research, and regenerative medicine. Naturally derived and synthetic hydrogels are good candidates for *in vitro* cell growth studies, owing to their soft and biocompatible nature; however, they are often hindered by limited ranges of stiffness and the requirement to modify the gel with additional extracellular matrix (ECM) proteins for cell adherence. Here, we report on the synthesis of a printable synthetic hydrogel based on cysteine-modified poly(acrylic acid) (PAA-Cys) with tuneable mechanical and swelling properties by incorporating acrylic acid into the PAA-Cys network and subsequent photoinitiated thiol-acrylate cross-linking. Control of the acrylic acid concentration and UV curing time produces a series of hydrogels with swelling ratios in excess of 100% and Young’s modulus values ranging from ∼2 to ∼35 kPa, of which most soft tissues fall within. Biocompatibility studies with RPE1 cells showed excellent cell adhesion and cell viability without the need for further modification with ECM proteins, but still can be modified as needed. The versatility of the hydrogel tuneable properties is demonstrated by culturing with RPE1 cells, which *in vivo* perform an important function in the visual process and the dysfunction of which may lead to various retinal abnormalities, such as glaucoma.

## Introduction

Cells in *in vivo* systems exist in a complex three-dimensional (3D) network of an extracellular matrix (ECM) composed of insoluble macromolecules such as collagens, fibronectin, elastin, and laminins. These macromolecules provide not only structural support for cell attachment but also external cues to drive cell differentiation, migration, and growth.^1−3^ In addition to insoluble ECM factors, cells are in constant contact with tissue-specific soluble growth factor gradients, which are also involved in cell survival, proliferation, and terminal differentiation.^3−6^

Furthermore, tissue topology has also been recognized as an important tissue-specific feature that supports cellular function, specifically in *in vivo* boundary cell layers, where many epithelial cells display a two-dimensional (2D) morphology.^7−9^ Importantly, the underlying superstructure has a distinct three-dimensional (3D) topology, which, along with localized ECM patterning, is also essential for robust niche dynamics. Indeed, in most epithelial environments, the topological arrangement of neighboring cells, driven in part by the bulk morphology of the environment, imparts biophysical cues that are an integral part of the normal niche homeostasis.^10−15^ Numerous studies have shown evidence of patterning in boundary layer cells according to topology, with various classes of cells, including stem cells, proliferating cells, and differentiated cells, self-organizing according to local 3D superstructural elements.^16−18^ This behavior is an important regulator of niche dynamics of boundary cells *in vivo* and within *in vitro* mimetic systems.^10−15^ For example, in *in vitro* studies, it was observed that epithelial cells cultured on substrates with pillar and pit architecture, similar to that of the intestinal crypt villus unit, experienced heterogeneous growth and distribution over a course of just 48 h.^8^ Similarly, while combining niche soluble factors, such as bone morphogenic proteins 2 (BMP-2) with a nanopatterned 2D array substrate, human mesenchymal stem cells showed enhanced osteogenic differentiation.^19^

Cumulatively, the unique and well-defined microenvironment of each tissue has been identified as one of the key drivers behind healthy tissue homeostasis.^20−22^ For instance, alterations of the topological environment of the underlying basement membrane (BM) in the retina have been reported as one of the leading causes of maculopathies.^23^ Here, evidence suggests that diseased BM shows minute variation in parameters such as stiffness, biochemical composition, porosity, and thickness, which all functionally impact various cell behaviors of the retinal pigmented epithelial layer (RPE).^23^ As even the most discrete changes in the microenvironmental features have been attributed to disease progression, namely, in the cases of cancer or other degenerative disorders such as glaucoma,^24−27^ there is a requirement for tuneable and biocompatible scaffolds, which would permit a more faithful recapitulation of these boundary layer environments.

*In vitro* cell culture models strive to mimic *in vivo* systems, with the common aim of cell culture studies being able to better understand and recapitulate *in vivo* cell behavior.^28^ Conventional cell culture studies are undertaken using uniform 2D structures such as plastic Petri dishes manufactured from hard plastics. Although these models are widely accepted by the scientific community, research shows that they do not accurately represent many *in vivo* physiological features and thus cannot fully mimic some cellular responses.^29^ Topological differences in tissue thickness or stiffness and overall morphology indicate region-specific cellular responses that cannot be accurately captured by conventional 2D culture.

Hydrogels have been identified as highly customizable platforms suitable to support cell cultures. Hydrogels are hydrophilic polymers that can hold many times their weight in water, making them similar to mammalian tissue in terms of morphology. Their hydrophilic and soft nature makes them ideal candidates for cell culture studies and possible tissue engineering applications.^30−32^ The biochemical and mechanical properties of hydrogels can also be varied or tuned, a distinct advantage given that such properties can influence cell behavior.^33,34^ Varying the stiffness of a substrate has been shown to alter stem-cell differentiation,^1^ while changing the proteins surrounding stem cells can drive cells from osteogenic to adipogenic lineages.^35,36^ The ability to control matrix stiffness is also advantageous while modeling diseased tissue where matrix stiffness exhibits the most alterations, for example, in glaucoma or cancer.^24−26^

Tuneable hydrogels for cell culture models may be synthesized from naturally derived or synthetic polymers. Naturally, derived hydrogels typically exhibit strong biocompatibility and cell adherence without the need for further modifications. However, they often display poor stability, mechanical properties, and have limited room for modification. Additionally, naturally derived hydrogels are potentially biologically active thereby reducing the control in the modeled environment and introducing experimental variations. Synthetic hydrogels are attractive alternatives for cell culture models as they can be tailored or modified to suit specific cell types.^37^ Additionally, synthetic hydrogels unlike naturally derived hydrogels do not have any associated biological activity, therefore providing a more controlled experimental environment. Poly(ethylene glycol) (PEG) and polyacrylamide (PA) are two widely investigated synthetic hydrogels, the mechanical properties of which can be easily tailored. However, they are both biologically inert and do not support cell adhesion and proliferation without additional modification to their surfaces with ECM proteins.^30^ As such, there is an indisputable need for biocompatible hydrogels that exhibit tuneable mechanical stiffness while simultaneously supporting cell adhesion, cell viability, and further modification with ECM as desired.

Unlike poly(ethylene glycol) and polyacrylamide, thiolated polymers such as cysteine-modified poly(acrylic acid) (PAA-Cys), are a class of synthetic polymers with excellent biocompatibility and cell adhesion properties. Pristine poly(acrylic acid) (PAA) although naturally biologically inert, once conjugated with cysteine (Cys), supports cell adhesion and exhibits excellent mucoadhesive properties. These PAA-Cys polymers, however, although they exhibit excellent biocompatibility, suffer from poor stability in aqueous solutions and begin to degrade after a few hours.^38,39^

This work documents an approach to synthesize PAA-Cys hydrogels with stable and tuneable mechanical properties for cell culture applications, especially those mimicking boundary layer cell environments. These can be used to model healthy or diseased tissues/organs, demonstrated through investigating RPE1 cell dynamics, which are particularly relevant to Bruch membrane-like environments. This is achieved by introducing acrylic acid as a copolymeric unit into the PAA-Cys network followed by cross-linking via a photoinitiated thiol-acrylate reaction. This cross-linking chemistry is advantageous because it provides high levels of spatiotemporal control, is insensitive to oxygen inhibition, and occurs under mild pH and temperatures.^40−45^ Mechanical properties and swelling behavior were modified by adjusting the UV exposure time and the ratio of AA to PAA-Cys content. Compression testing and swelling studies were carried out to ascertain the degree to which these properties could be modified. Cell viability and proliferation studies using normal retina pigment epithelial (RPE1) cell lines were performed to ascertain the effect of the copolymer composition and hydrogel elasticity on cell adhesion and cell proliferation rates. Finally, the effects of incorporating a bioactive protein into the hydrogels during cell culture studies were investigated. Therefore, we feel that the tuneable nature of the hydrogel system we report here is consistent with its potential application in generating a 3D topological substrate with micropatterning region-specific properties that can act as suitable 2D substrates to investigate boundary layer cell dynamics.

## Experimental Section

### Materials

Poly(acrylic acid) (PAA) (450 kDA), acrylic acid (AA), and 2-hydroxy-4′-(2-hydroxyethoxy)-2-methylpropiophhenone (Irgacure 2959) were purchased from Sigma-Aldrich (Ireland). l-Cysteine hydrochloride was purchased from Alfa Aesar (Ireland). 1-Ethyl-3-(3-dimethylaminopropyl)carbodiimide (EDC) was purchased from Tokyo Chemicals (United Kingdom). All other chemicals used were analytical grade and were used without further purification.

### Synthesis of Tuneable Hydrogels

#### Synthesis of Thiolated PAA

The PAA-Cys hydrogels were synthesized from a method adapted from Iqbal et al.^39^ Overall, 1 g of PAA 450 kDa was dissolved in approximately 100 mL of distilled water. The pH of the solution was adjusted to approximately pH 5 with 5 M NaOH. Next, 2 mL of EDC was diluted in 10 mL of distilled water and subsequently, the pH was adjusted to approximately pH 5 using 5 M HCl. EDC was added dropwise to the solution of PAA over a period of 20 min. The reaction was stirred at room temperature for an additional 10 min. A total of 1 g of l-cysteine hydrochloride was dissolved in 10 mL of distilled water, and the pH adjusted to approx. pH 5 with 5 M NaOH. The solution of l-cysteine hydrochloride was added slowly to the solution of PAA and EDC. The pH of the final solution was adjusted to approx. pH 5.5. Nitrogen was purged through the reaction for 1 min, after which the reaction vessel was allowed to stir at room temperature for 3 h. The reaction mixture was dialyzed five times using a Spectra/Por membrane (MWCO: 1200) at low pH conditions. The solution was then lyophilized at reduced pressure and temperature. The composition of the thiolated PAA was confirmed using Fourier transform infrared spectroscopy (FITR). FTIR spectra were collected using a Nicolet FTIR spectrometer.

#### Hydrogel Synthesis

Hydrogels were fabricated by photo-cross-linking PAA-Cys with acrylic acid at differing concentrations. A stock solution of a 5% (w/v) initiator was prepared by dissolving Irgacure 2959 in a solution of 1:1 water and ethanol. A precursor hydrogel solution was formed from a 2% solution of PAA-Cys and acrylic acid mixed at the predetermined ratios. Overall, 0.3% (w/v) of Irgacure 2959 was added to the solutions followed by exposure to UV light at a wavelength of 365 nm for a range of predetermined times. For simplicity, all ratio calculations were made relative to the PAA-Cys solution, and their names were abbreviated accordingly as PCA1, PCA4, and PCA7, respectively.

#### Structural Characterization

FTIR and scanning electron microscopy (SEM) analyses were carried out on the samples to determine the chemical and physical properties of the hydrogels. SEM micrographs were obtained on a JOEL Carry Scope JCM-5700. FTIR spectra were collected using a Nicolet IS50 FTIR spectrometer.

#### Compression Testing

Compression testing was carried out on a Mecmesin MultiTest 2.5 dV. Test samples were prepared by pipetting approximately 1 mL of the hydrogel precursor solution containing the photoinitiator in a 12-well plate. Samples were then exposed to UV light at 365 nm, for the predetermined times outlined in Table 1. Following this, the samples were swollen in water for 48 h prior to testing.

**Table 1:** Parameters for Hydrogel Synthesis^a^

parameters	PAA-Cys	acrylic acid	Irgacure 2959	wavelength (nm)	exposure time (min)
PAA-Cys-1AA (PCA1)	2% w/v	1.1% v/v	0.3% w/v	365	10
					20
					30
PAA-Cys-4AA (PCA4)	2% w/v	4.2% v/v	0.3% w/v	356	10
					20
					30
PAA-Cys-7AA (PCA7)	2% w/v	7.4% v/v	0.3% w/v	356	10
					20
					30

a Three-hydrogel compositions, each with three different cross-linking times, resulting in a total of nine hydrogels synthesized.

Constrained compression testing took place using a 19 mm plate. Hydrogels were compressed at a strain rate of 0.8 mm/min. Force and displacement data were collected converted into stress–strain curves. All measurements were carried out in triplicate.

#### Swelling Ratio

The equilibrium water content of the hydrogels was determined by the weight ratio of the swollen polymers in water. The swelling ratio of the thiolated PAA was found using the equation
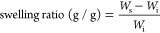
where *W*_s_ and *W*_i_ are the weights of the swollen hydrogel and the initial, dry polymer, respectively. All measurements were replicated in triplicate.

#### Hydrogel Suitability Evaluation for *In Vitro* Applications

Hydrogel suitability for *in vitro* cell culture was evaluated using an immortalized retina pigment epithelium cell line (RPE1) (CRL-4000, ATCC). RPE1 cells were allowed to grow on both collagen or phosphate-buffered saline (PBS) swelled hydrogels under normal culture media conditions for 72 h. Hydrogel preparation and cell culture conditions are outlined in the proceeding sections.

#### Hydrogel Preparation for Cell Culture

Following polymerization, hydrogels were sterilized with 20 min of UV exposure under sterile laminar flow hood conditions. Afterward, hydrogels were swelled at room temperature for 2 h in either PBS or a collagen solution diluted with PBS to a final concentration of 25 mg/mL (C3867, Sigma-Aldrich Inc.). To remove acidic byproducts formed during hydrogel polymerization, gels were washed three times with Dulbecco’s modified Eagle medium (DMEM) (D6421, Sigma-Aldrich Inc.) or DMEM containing 25 mg/mL collagen, for PBS or collagen swelled conditions, respectively.

#### Cell Culture

Prior to seeding on hydrogels, RPE1 cells were grown under normal cell culture conditions at 37 °C and 5% CO_2_ in DMEM supplemented with 10% fetal bovine serum (Sigma-Aldrich Inc.), 1% penicillin/streptomycin (Sigma-Aldrich Inc), and 1% GlutaMAX (35050061, ThermoFisher Scientific) until they reached 80% density. Afterward, cells were trypsinized (T4049, Sigma-Aldrich Inc.), counted, and diluted to a concentration of 20 000 cells/mL.

The excess liquid left after swelling was aspirated from the hydrogel surface and 1 mL of normal RPE1 growth media containing 20 000 RPE1 cells were dispensed into each well. Cells seeded on hydrogels were cultured for 72 h at 37 °C and 5% CO_2_ in DMEM normal culture media described in the preceding section.

#### Cell Cytotoxicity

Cytotoxicity was evaluated after 72 h of cell culture, using a live and dead cell assay kit (ab115347, Abcam). Briefly, media were aspirated from each well and replaced with live and dead cell assay solution diluted in RPE1 cell culture media to a final concentration of 1X as per the manufacturer’s instructions and incubated in the dark for 10 min at room temperature. Subsequently, the live and dead cell assay solution was aspirated and replaced with Hoechst 33342 (B2261, Sigma-Aldrich) diluted at 1:10 000 in cell culture media. Cells were further incubated for 5 min, after which the solution was aspirated from each well. Wells were washed three times with PBS before being replaced with culture media for the duration of the imaging. Plates were immediately imaged with an ImageXpress Micro Confocal High-Content Imaging System (Molecular Devices). Overall, 16 fields of view repeated in triplicate were used for subsequent quantification, with each image set, equivalent to one field of view/site composed of three fluorescent channels. Obtained images were quantified using open source CellProfiler software using a variety of image processing protocols developed in house. Numeric image analysis outputs were statistically analyzed applying one-way ANOVA with Bonferroni’s multiple comparison test; a significance of *p* < 0.05 was reported. Data generated are shown in Figure 5A,C.

## Results and Discussion

### Hydrogel Synthesis

Nine PAA-Cys-co-acrylic acid (PCA) hydrogels were synthesized in this study. Table 1 outlines the constituent ratios of each component and corresponding UV exposure times. For cell culture applications, a thorough understanding of factors influencing the hydrogel mechanical properties is essential, as scaffold mechanical strength is a key influencer of cell behavior *in vivo*. Previous studies on thiol-acrylate-based polymers have shown that the ratio of thiol to acrylate functional groups significantly influences the polymer mechanical properties. Similar trends were observed by Park et al. and Wu et al. who reported that after a certain degree of thiolation, the hydrogels showed no changes in mechanical properties.^46,47^ Results suggest that there is an equilibrium point in the PCA hydrogels after which the additional thiol concentration does not influence cross-linking conversion rates and may have adverse effects on the hydrogel properties. Thus, in this study, the thiol content was held constant by maintaining the concentration of a PAA-Cys polymer while the acrylate content was varied. Findings also suggest that the cure time affects the mechanical and swelling properties of the hydrogels to some degree. However, the ratio of acrylate to the thiol content is a key influencing factor on final hydrogel properties, with hydrogels, showing more prominent mechanical and swelling property changes in response to varying reactant concentrations.

**Figure 1:**
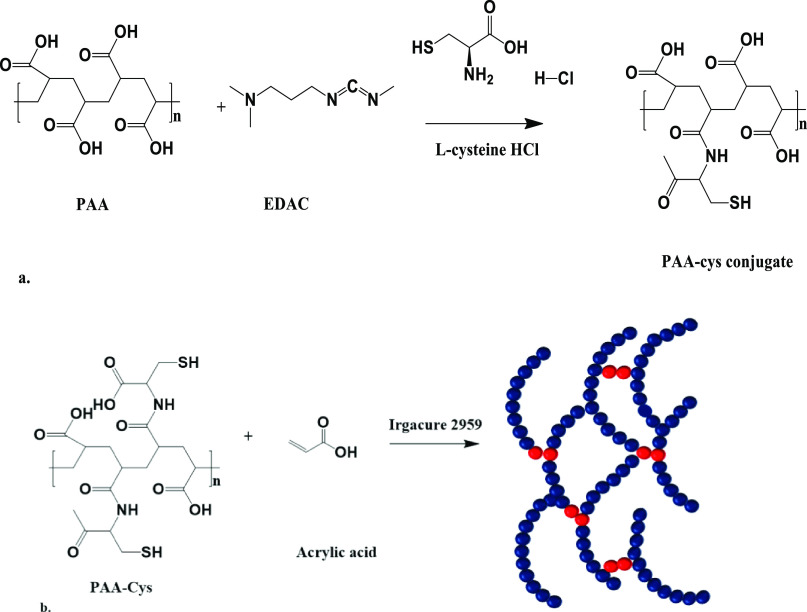
Schematic of synthesis of (a) PAA-Cys polymer and (b) PAA-Cys-AA hydrogels.

FTIR was used to confirm the chemical structure of the parent PAA-Cys polymer used in the synthesis (Figure S1) and the subsequent hydrogel formed after UV cross-linking. As the hydrogel consists of acrylic acid and thiolated PAA, it is expected to have carboxylic acid functionalities, amide groups, and disulfide bonds (Figure 1).

**Figure 2:**
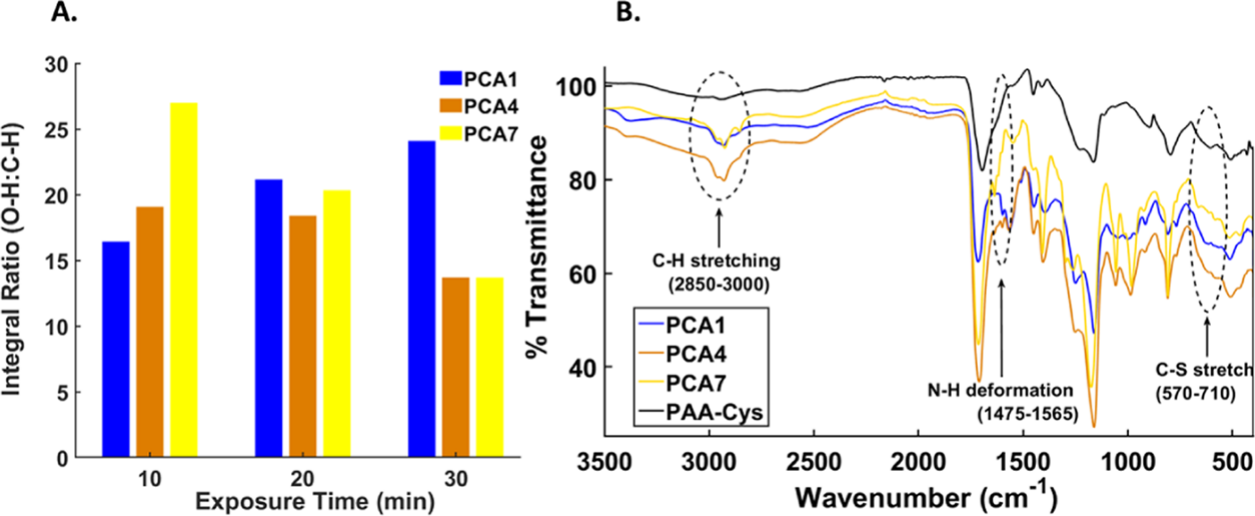
(A) Representative plots showing the ratio of OH:CH from the samples with varying concentrations and exposure times and (B) representative FTIR spectra overlay showing an increase in the C–H stretching between 2850 and 3000 cm^–1^.

Figure 2B shows FTIR spectra of the PAA-Cys polymer and a selection of the PCA hydrogels. The absence of a thiol stretch between 2500 and 2600 cm^–1^ is vital in confirming the formation of the hydrogels (Figure S1). There are two competing cross-linking mechanisms occurring during the formation of the hydrogels, namely, thiol-acrylate cross-linking and cross-linking of the PAA-Cys backbone. The disappearance of the −SH peak in the spectra is associated with the thiol-acrylate reaction and corresponds to the consumption of thiol groups during the cross-linking reaction.

The PAA component of the PAA-Cys backbone can also cross-link in the presence of UV light to form an insoluble hydrogel and has been observed when pure PAA polymers undergo photo-cross-linking. It is theorized that the process occurs via the formation of a radical on the carbon atoms bearing the −COOH functionality.^48,49^ These radicals can then recombine, forming C–C bonds thus effectively cross-linking the polymer. This form of cross-linking results in a reduction of the COOH groups present and an increase in the CH groups for each sample. The extent to which this form of cross-linking is occurring in the PCA hydrogels can also be assessed by FTIR. The integral of the area under the peak between 3700 and 2400 cm^–1^ corresponds to the concentration of −OH adjacent to the carbonyl group, while the narrow peak between 3000 and 2850 cm^–1^ corresponds to the CH stretch of an alkane. Figure 2A shows the integrated area for each peak relative to one another, permitting the changing ratios of OH/CH to be observed as function of UV exposure.

Results showed a decrease in the ratios of O–H relative to C–H groups in PCA4 and PCA7 hydrogels as UV exposure increases, thereby confirming the consumption of COOH groups during hydrogel synthesis. An opposite effect is observed for PCA1 hydrogels; however, this may be attributed to reduced cross-linking across the structure due to the low concentration of AA in the precursor when compared to PCA4 and PCA7.

### Mechanical Properties of the Hydrogel

**Figure 3:**
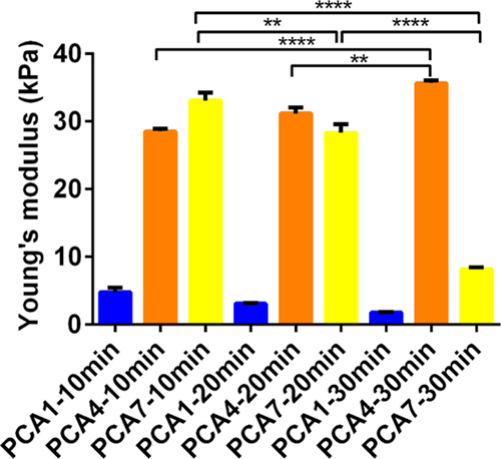
Hydrogel stiffness obtained from compression testing results showing Young’s modulus values obtained for the different hydrogel compositions formed at different times (error bars, mean ± SD, *n* = 3).

Compression testing is one of the ways in which the mechanical properties of a soft material can be probed. The stiffness of a material is measured through resistance to deformation in response to applied force, which is also referred to as Young’s modulus. Each of the hydrogels fabricated underwent confined compression testing to assess mechanical properties. Stress–strain curves for all of the hydrogels were generated and the linear portion of the curves was used to determine Young’s modulus. Figure 3 shows Young’s modulus for each respective hydrogel. Hydrogel stiffness ranged from approximately 2–35 kPa, with elastic moduli values lying on the lower end (∼1.6 to ∼9 kPa) and the higher end (∼25 to 35 kPa) of that range. It should be noted that the elastic modulus typically lies between 0.1 and 10 kPa for soft tissue and anywhere upward of 20 kPa for stiffer tissues.^50−52^ Brain tissue has an elastic modulus of several hundred Pascals, whereas tendon and cartilage have modulus values in the mega Pascal range.^53^ The elastic moduli of some cell types, such as retinal epithelial cells, cover a large range; in this case, ranging from 0.3 to 125 kPa, as values change in response to age and health.^54−57^

For maximum cellular growth and proliferation, any substrate used for cell culture studies should possess physical properties that can be tailored for the specific tissue type or cell environment. Mechanical testing of the PCA hydrogels show it is possible to produce a range of modulus values by simply altering the acrylic acid concentration. The overall mechanical strength of the hydrogels is influenced by two processes, namely, covalent cross-linking, and the formation of polymeric branching. The thiol radical formed in the presence of a photoinitiator and UV light reacts with the acrylate group forming intra- and intermolecular covalent bonds across the polymer, leading to a cross-linked and stable structure. The same mechanism leads to the formation of PAA branches anchored to PAA-Cys polymer backbones that also influence mechanical properties. The PCA1 sample contained the least ratio of acrylate to thiol groups, thereby yielding the lowest Young’s modulus values at all exposure times.

Effects of UV exposure time on hydrogel mechanical strength were also investigated by examining hydrogel mechanical strength at three different UV exposure times. According to the principles of photo-cross-linking, an increase in the exposure time leads to an increase in cross-links formed, resulting in a stronger hydrogel. However, in two of the hydrogels, an opposite trend is observed (Figure 3). PCA1 and PCA7 exhibit a higher Young’s modulus at a lower exposure time and a lower value at a higher exposure time. This can be attributed to polymer degradation previously reported for PAA polymer systems. When PAA-Cys is cross-linked using UV light, the formation of covalent cross-links between thiol and acrylic acid moieties is accompanied by PAA polymer degradation via β-scission. This degradation affects the PAA molecules involved in covalent cross-linking of the thiol groups and PAA branching, the two critical factors influencing hydrogel mechanical strength. One of the degradation products is an acyl radical, which can recombine to form the parent polymer.^58^ PCA1 has the lowest v/v ratio of AA to PAA-Cys in the hydrogel precursor and as such exhibits less cross-linking and PAA branching. The low levels of cross-linking and PAA branching ensure mechanical properties of PCA1 are impacted by β-scission degradation induced by increased UV exposure. Similar trends are observed in PCA7 because of similar mechanistic complexities; however, in this case, it is enhanced due to increased concentrations of AA. PAA degradation via β-scission is very efficient and occurs at a faster rate at lower pHs.^48,59^ As PCA7 contains the highest volume of AA in the hydrogel precursor, it has a lower pH and is more prone to this process relative to other hydrogels. While the β-scission degradation also occurs in PCA4 at higher UV exposure times, there are sufficient concentrations of AA to maintain hydrogel mechanical strength via cross-linking and branching, yet insufficient AA concentrations to induce enhanced PAA β-scission degradation. Results indicate that varying the AA concentration is the preferred method for synthesizing hydrogels with elastic moduli ranging from ∼1.6 to 35 kPa. However, inducing β-scission degradation of the PAA component within the hydrogel postsynthesis represents an interesting approach to altering cell scaffold strength postculturing to produce a truly dynamic and responsive scaffold.

### Surface Morphology

**Figure 4:**
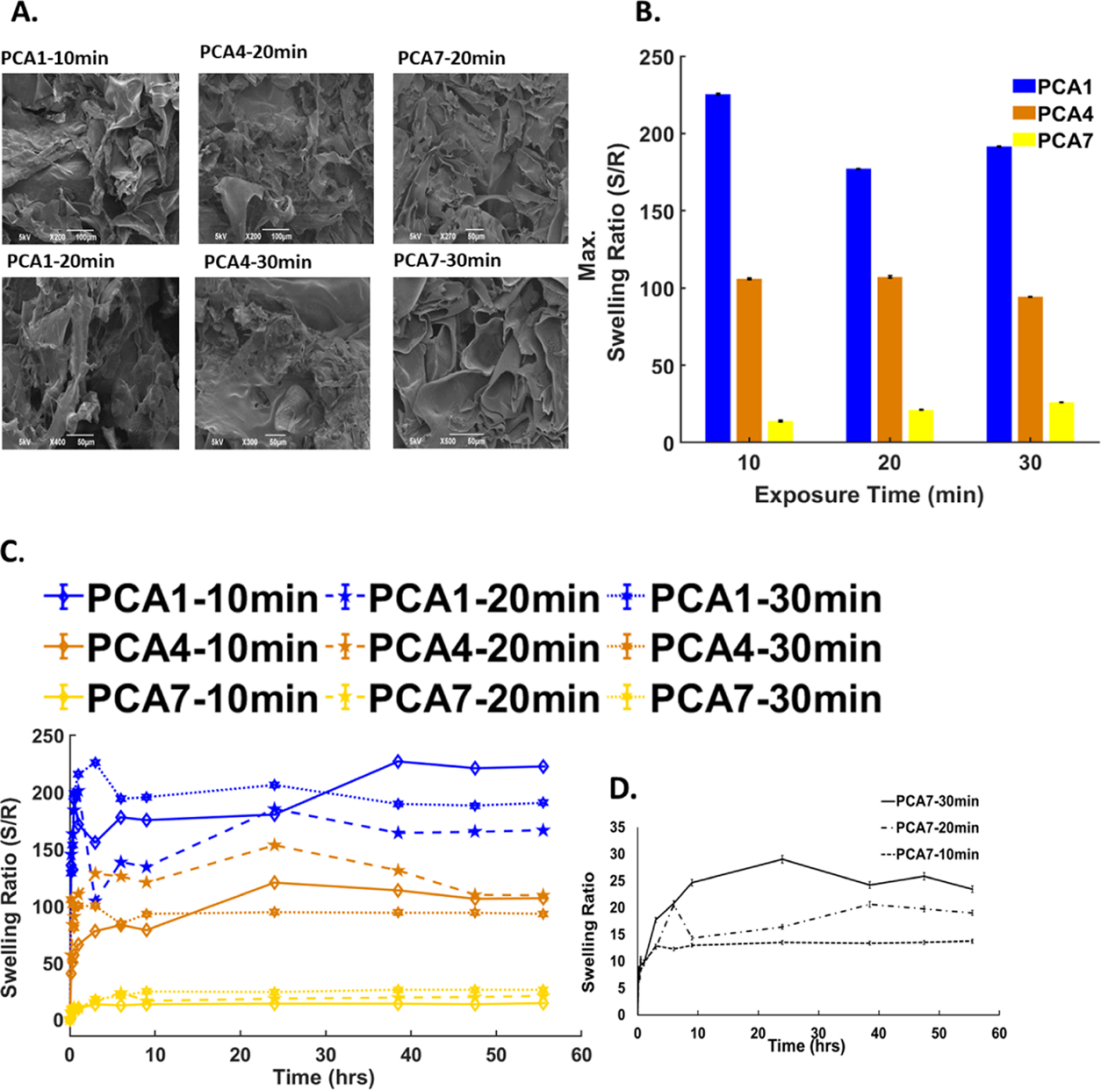
(A) SEM micrograph of hydrogels formed, (B) equilibrium swelling ratio of the three-hydrogel compositions at their respective exposure times, (C) graphical representation of swelling ratio of PCA7 hydrogels over 24 h, and (D) graphical representation of the swelling ratio of all nine hydrogels over 24 h (error bars, mean ± SD, *n* = 3).

Surface morphology and topography have previously been shown to influence cell adhesion and as such the surface morphology of the PCA hydrogels was examined by scanning electron microscopy (SEM). Figure 4A shows the SEM images of the freeze-dried hydrogels after swelling. Hydrogel morphology appeared broadly similar, indicating that UV cross-linking time does not appear to have a significant effect on surface morphology. All hydrogels exhibited what appeared to be a stacked or layered surface consisting of roughened and smoothed areas when samples were viewed from the sliced cross-sectional area. This is most likely a result of the copolymeric nature of the hydrogels. The stacked nature of the samples may also favor the formation of porous layers during swelling.

### Hydrogel Swelling Behavior

The swelling ratio has a direct correlation with the chemical structure and mechanical properties of a hydrogel. A higher swelling ratio is typically an indicator of a softer gel, which is an indicator of decreased cross-linking. Figure 4B shows the maximum swelling ratios for each of the gels as a function of UV exposure time. The PCA1 hydrogels exhibited the highest swelling ratio and a capacity to absorb over a hundred times their weight in water. PCA4 exhibited roughly half the swelling ratio of PCA1, while PCA7 exhibited the lowest swelling ratio of each of the materials examined. The swelling ratios correlate with the results observed during mechanical testing as the hydrogel with the lowest elastic modulus exhibited the highest swelling ratio. The effect of UV curing times on hydrogel swelling ratios was also examined. The PCA1 and PCA4 samples cured for 10 min showed the highest maximum swelling capacity, whereas samples cured for 20 and 30 min exhibited slightly lower maximum swelling capacity values. In PCA7, however, samples cured for 30 min exhibited the highest swelling ratio, and the samples cured for 20 and 10 min showed significantly lower swelling ratios. This is likely due to polymer degradation due to the enhanced β scission degradation as a result of increased AA concentrations similar to the trends observed during mechanical testing studies.

The rate at which hydrogels absorb water is an important criterion in determining suitable applications. Figure 4C,D shows the time-dependent swelling studies for each of the respective hydrogels. All samples exhibited an initial elevated intake of water followed by a loss and then a rise and plateau. Similar trends have previously been observed for PAA-Cys polymers for drug delivery applications. PCA1 and PCA4 exhibit a rapid swelling rate, while PCA7 displays a more gradual swelling rate. Although both UV curing time and AA concentration have been shown to influence hydrogel mechanical strength, results show that the AA concentration is the most significant factor affecting swelling rate. All hydrogels reached equilibrium swelling in under 5 h. Unlike previously reported PAA-Cys hydrogels that are stable for a period of 2–3 days, all samples were stable for a minimum of 2 weeks with no hydrogel disintegration being observed during this time. PCA4- and PCA7-based hydrogels exhibited stability for over 3 weeks. Medium to long-term stability is a key requirement of synthetic hydrogels for cell culture and cell scaffolds.

### *In Vitro* Cell Study

**Figure 5:**
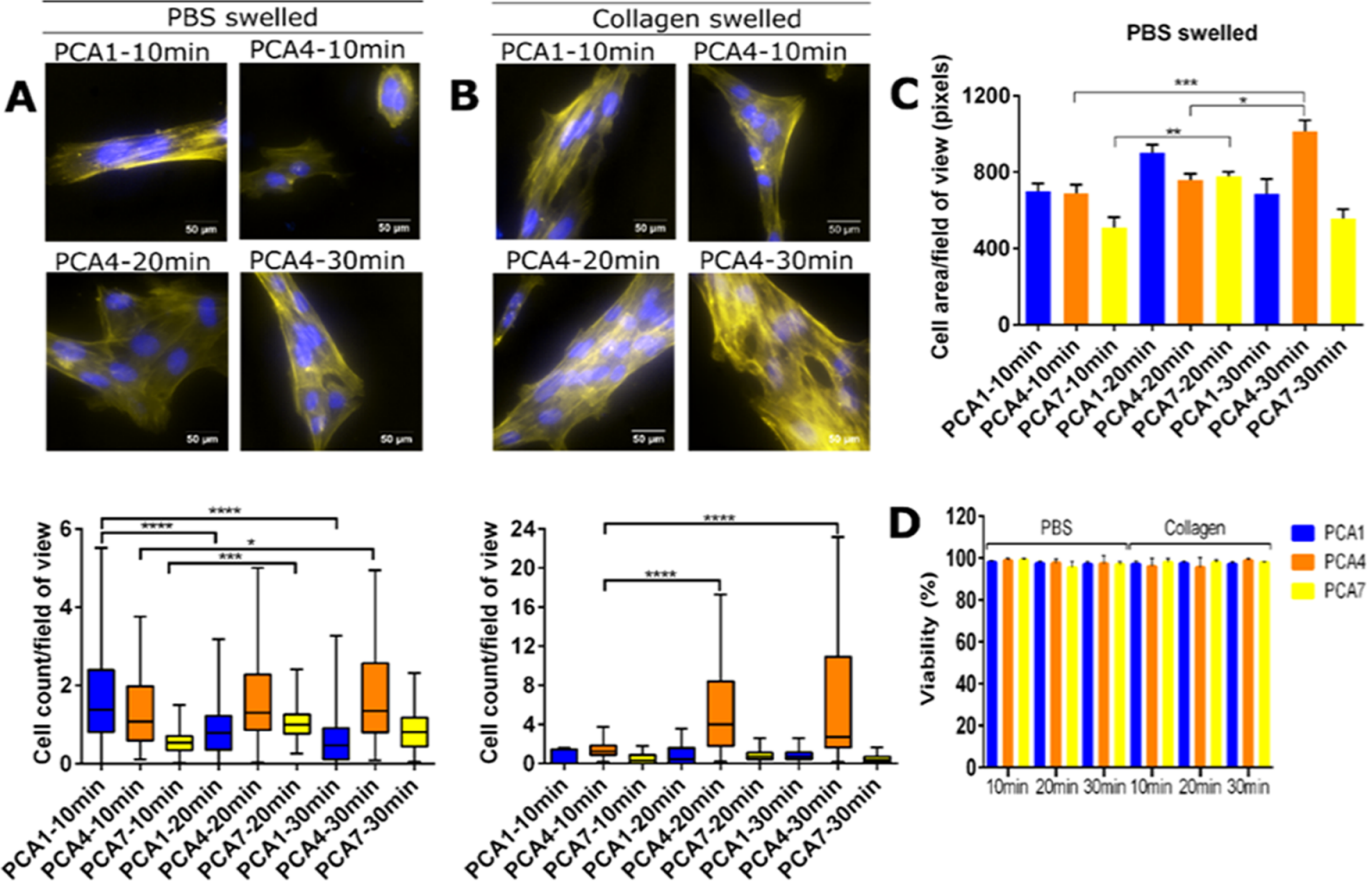
(A) Whisker plots representing cell count per field of view of the PBS swelled conditions and images of hydrogels with greatest cell growth, where yellow is actin and blue is DAPI/DNA (scale bar represents 50 μm). (B) Whisker plots representing cell count per field of view of the collagen swelled conditions and images of hydrogels with greatest cell growth, where yellow is actin and blue is DAPI/DNA (scale bar represents 50 μm). (C) Cell area in pixels/field of view in the PBS swelled condition. (D) Percentage cell viability, where hydrogels are grouped by their curing times (e.g., 10, 20, and 30 min).

Each of the nine hydrogels exhibited high levels of biocompatibility when used as cellular growth supporting scaffolds for RPE1 cells, the hTERT-immortalized retinal pigment epithelial cell line that exhibits normal growth dynamics. Each hydrogel displayed greater than 98% biocompatibility when assayed by confocal microscopy using a live-dead fluorescent analysis technique (Figure 5D).

Interestingly, marked differences in RPE1 cell proliferation were observed. These differences predominantly coincide with increasing or decreasing hydrogel mechanical strength attributed to the joint influence of composition and curing time. Overall, hydrogels swelled with PBS, PCA1-10 min, PCA4-10 min, PCA4-20 min, and PCA4-30 min represented in Figure 5A, showed the highest degree of cell proliferation. In the presence of these hydrogels, cells tended to stretch normally with actin bundle stress fiber formation, which is traditionally associated with nonmuscle cells, and are known to play an important role in cell adhesion, migration, and morphogenesis events.^60^ Furthermore, these features demonstrate that functional focal adhesions indicative of robust cellular attachment that were resistant to actomyosin tension were created.^61^

As anticipated, there are cell proliferation differences, both intra- and intergroup, corresponding to the effects of hydrogel composition and different curing times on the mechanical strength of these hydrogels. For example, PCA1 cell proliferation decreased with increased curing time, which although nonsignificantly (Figure 3) lowered the mechanical strength from 5 kPa at 10 min, to 3 kPa at 20 min, and to 2 kPa at 30 min, possibly missed the optimal physiological stiffness window for this cell lines’ active proliferation. Similarly, a study conducted by Hadjipanayi et al. revealed decreased cell cycle rates of dermal fibroblasts cultured on more compliant collagen matrices, compared to stiffer gels of the same base material.^62^ PCA4, however, showed a statistically significant increase in mechanical strength attributed to longer curing times: 29, 31, and 36 kPa for 10, 20, and 30 min, respectively (Figure 3). Gradual mechanical strength increase in PCA4 due to longer curing times provided a suitable substrate for enhanced cell proliferation and spreading (Figure 5A–C). In the case of PCA7, the significantly decreasing mechanical strength with increased curing times did not have any significant effects on cell proliferation and mildly significant effects on cell spreading in the case of 10 and 20 min curing times. This could perhaps be attributed to an initial nonoptimal hydrogel chemical composition.

Furthermore, hydrogels were evaluated for suitability of active ECM biomolecule incorporation. Collagen was selected as an active biomolecule due to its abundance in the body and known influence on cell proliferation and adhesion.^63,64^ When hydrogels were swelled with PBS containing 25 mL/mL collagen in some instances, overall cell proliferation levels increased (Figure 5B), indicating that collagen was available for cell interaction. For example, the most significant effects were observed in PCA4 (−20 min) and PCA4 (−30 min), where cell proliferation for both was approximately 3.5-fold increased in collagen swelled hydrogels compared to PBS. Interestingly, PCA1- and PCA7-based hydrogels showed negligible differences in cell proliferation when compared to the samples swelled in PBS.

All hydrogels swelled with PBS or collagen showed excellent biocompatibility and suitability to be used as a support material for cellular growth without the need for further modification designed to promote cell adhesion or enhance viability. Additionally, collected data indicate that bioactive molecules can be readily incorporated into synthetic hydrogels as a further customizable parameter for cell growth studies.

## Conclusions

The synthesis of PAA-Cys-AA-based hydrogels with readily tuneable mechanical and swelling properties and enhanced stability was reported. Hydrogel elasticity could be tuned by varying the photo-cross-linking time and the reactant concentrations to produce a range of elastic moduli similar to some healthy or diseased mammalian tissues. In the case of RPE1 cells used in this study, the use of these hydrogels has the potential to model glaucoma or other degenerative disorders affecting the ocular system components or even beyond that to model gastrointestinal system tissues, such as intestinal mucosal epithelium, where cells essentially grow in 2D along the crypt villus unit.^3^ The hydrogels formed had excellent swelling properties, exceeding 100% in each case, and were found to be highly biocompatible during evaluation with RPE1 cell lines. Significantly, the modified PAA-Cys hydrogels supported cell adhesion without the need for surface modification, and their mechanical properties were seen to directly influence cell proliferation. Hydrogels could be functionalized with bioactive ligands thereby making them suitable for specific protein encapsulation or surface coating. Owing to their printability, tuneable nature of their mechanical and swelling properties and their subsequent effect on cell behavior, these hydrogels allow for future fabrication of architecturally, biochemically and mechanically tailored cell and tissue scaffolds with a wide range of modifiable properties including varied topology and patterning as are seen in boundary cell niche environments.

This study is associated with a few inherent limitations. Hydrogels presented in this study were not tested for long-term stability, which is often needed while undertaking long-term cell culture experiments.^65−67^ Likewise, PAA-Cys-AA hydrogels are not amenable to biodegradation by cells, which does not allow resident cells to remodel their own environment or to penetrate into the gel environments, which may be an advantageous feature if these scaffolds would be used in the field of regenerative medicine.

This study provides a “tool box” platform of printable hydrogels that can be functionalized with a selection of biomolecules and proteins, as the current literature offers increasing evidence of such biomolecule importance in the modulation of various tissues homeostasis.^3^ Due to the high levels of PAA-Cys-AA hydrogel biocompatibility, they can also be implemented in the targeted drug delivery applications, where these hydrogels could serve as drug carriers.^68,69^ Particularly, cancer treatments could benefit from localized drug exposure.^69^ The highly tuneable stiffness of these hydrogels offers a possibility to cover a range of tissues and cells in physiological environments, opening the doors to future applications in tailored regionally localized tissue engineering applications, such as vascular or barrier tissue engineering.^70−72^

## Supporting Information Available

FTIR image of the PAA-Cys-AA outlining amide formation during the cross-coupling reaction (PDF)
