# Supplementary material for: Synthesis of Poly(acrylic acid)-Cysteine-Based Hydrogels with Highly Customizable Mechanical Properties for Advanced Cell Culture Applications
Source: ACS Omega. 2022 Mar 11;7(11):9108–17. doi: 10.1021/acsomega.1c03408 (PMC8945188; doi:10.1021/acsomega.1c03408)
Supplement: Supplementary file 1 — ao1c03408_si_001.pdf [file ao1c03408_si_001.pdf]

Supporting Information

Synthesis of Polyacrylic-acid-Cysteine based Hydrogels with highly customisable mechanical properties for advanced cell culture applications

Sharon O Bolanta[1], Sigita Malijauskaite[1], Kieran McGourty[1,2,], Emmet J O'Reilly[1,#]

[1]Dept. of Chemical Sciences, [2]Bernal Institute University of Limerick, Limerick, Ireland.

[2]Health Research Institute (HRI), University of Limerick, Limerick, Ireland.

[#]Corresponding author: Dr Emmet O'Reilly, Bernal Institute, emmet.oreilly@ul.ie

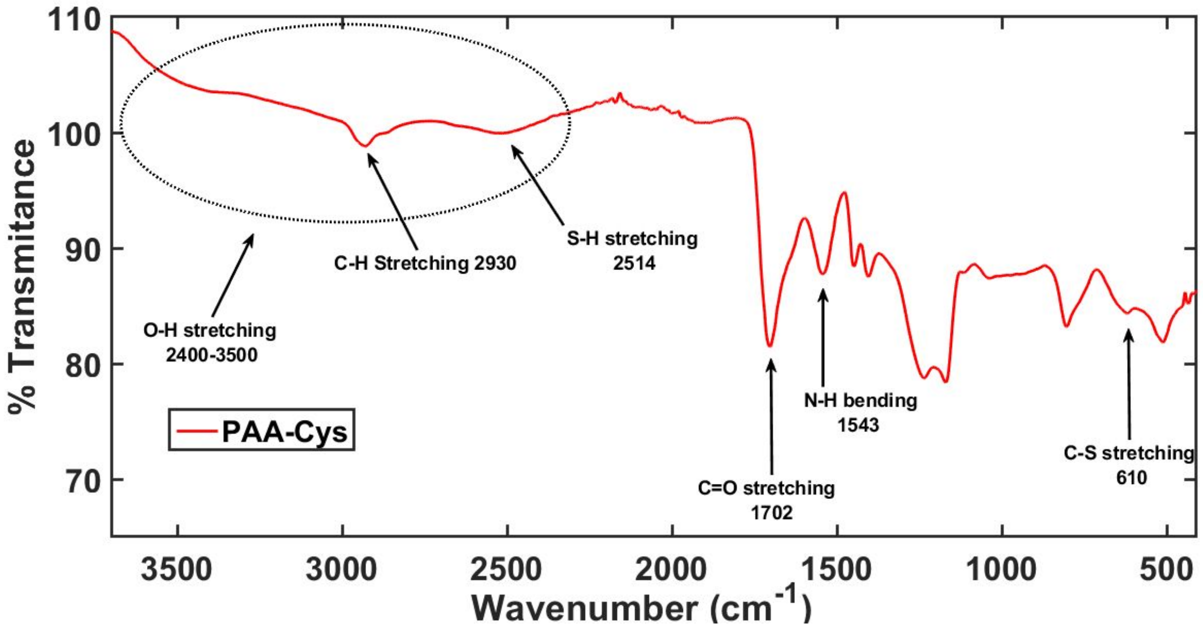

Figure S1; *FTIR spectra of PAA-Cys highlighting relevant peaks.*

A strong peak at ~1545 cm^{-1} confirmed the presence of the NH group in secondary amides. The amide formation is the building block of the thiolated polymer as it the bond which joins the carboxylic acid of the PAA to the cysteine molecule. This is formed during the coupling reaction of poly(acrylic acid) to cysteine. A weak peak at ~2514 cm^{-1} confirmed the presence of thiol groups, and the peak at 1700 cm^{-1} confirmed the presence of the carboxylic acids. These peaks were present in the thiolated polymer confirming the coupling of cysteine to poly(acrylic acid).
